# The Effect of Three Different (-135°C) Whole Body Cryotherapy Exposure Durations on Elite Rugby League Players

**DOI:** 10.1371/journal.pone.0086420

**Published:** 2014-01-29

**Authors:** James Selfe, Jill Alexander, Joseph T. Costello, Karen May, Nigel Garratt, Stephen Atkins, Stephanie Dillon, Howard Hurst, Matthew Davison, Daria Przybyla, Andrew Coley, Mark Bitcon, Greg Littler, Jim Richards

**Affiliations:** 1 Allied Health Research Unit, University of Central Lancashire, Preston, United Kingdom; 2 AHRu, University of Central Lancashire, Preston, United Kingdom; 3 Institute of Health and Biomedical Innovation, Queensland University of Technology, Kelvin Grove, Queensland, Australia; 4 SSTO, University of Central Lancashire, Preston, United Kingdom; 5 SENS, University of Central Lancashire, Preston, United Kingdom; 6 Wigan Warriors Rugby League, Central Park, Montrose Avenue, Wigan, United Kingdom; The Ohio State University, United States of America

## Abstract

**Background:**

Whole body cryotherapy (WBC) is the therapeutic application of extreme cold air for a short duration. Minimal evidence is available for determining optimal exposure time.

**Purpose:**

To explore whether the length of WBC exposure induces differential changes in inflammatory markers, tissue oxygenation, skin and core temperature, thermal sensation and comfort.

**Method:**

This study was a randomised cross over design with participants acting as their own control. Fourteen male professional first team super league rugby players were exposed to 1, 2, and 3 minutes of WBC at −135°C. Testing took place the day after a competitive league fixture, each exposure separated by seven days.

**Results:**

No significant changes were found in the inflammatory cytokine interleukin six. Significant reductions (p<0.05) in deoxyhaemoglobin for gastrocnemius and vastus lateralis were found. In vastus lateralis significant reductions (p<0.05) in oxyhaemoglobin and tissue oxygenation index (p<0.05) were demonstrated. Significant reductions (p<0.05) in skin temperature were recorded. No significant changes were recorded in core temperature. Significant reductions (p<0.05) in thermal sensation and comfort were recorded.

**Conclusion:**

Three brief exposures to WBC separated by 1 week are not sufficient to induce physiological changes in IL-6 or core temperature. There are however significant changes in tissue oxyhaemoglobin, deoxyhaemoglobin, tissue oxygenation index, skin temperature and thermal sensation. We conclude that a 2 minute WBC exposure was the optimum exposure length at temperatures of −135°C and could be applied as the basis for future studies.

## Introduction

Whole body cryotherapy (WBC) is the therapeutic application of extremely cold dry air, usually between −110°C and −140°C [1], [2]. WBC is becoming popular amongst athletes, coaches and clinicians across a variety of sports in order to prevent injury and promote recovery. Typically the timescale of exposure is reported as between 2–3 minutes [2]. Although a number of studies [1], [3]–[8] have investigated the physiological effects of WBC, the optimal WBC protocol required to initiate beneficial physiological responses is unknown [2], [9]. This is due to the lack of randomised controlled clinical studies investigating either exposure duration or number of treatment cycles [2], [9]. One of the more commonly reported beneficial changes following WBC is a reduction in inflammatory markers [2], [10], [11], [12]. Recently the acceleration of recovery from exercise induced muscle damage (EIMD) was reported after three WBC exposures [13]. However Costello and colleagues [1] reported ineffective results of WBC when administered 24 hours following eccentric exercises in order to help alleviate muscle soreness. Furthermore the enhancement of muscle force recovery was demonstrated to be ineffective in this study [1], [3]. One previous study has evaluated various physiological changes in professional rugby players, demonstrating changes in haematological profiles, following a protocol of 5 WBC exposures over 5 consecutive days [14]. However this study lacked a control group, therefore it is difficult to discriminate WBC effects compared to the cumulative effects of training. Rugby league is a contact sport, with a match consisting of two, forty minute halves of play where players engage in high intensity activity, intermittent with periods of low activity [15]. Due to the physical demands placed upon rugby league players which result in symptoms of fatigue and muscle performance decrements [15], WBC has recently become very popular as a recovery modality within this sport. The purpose of this study was to determine optimal WBC exposure duration and to measure physiological and perceptual responses following a 1, 2 and 3 minute WBC exposure at −135°C in elite rugby league players the day after a competitive fixture.

## Materials and Methods

### Ethics Statement

The study was conducted according to the Declaration of Helsinki (WMA, 2008), was approved by UCLan Built, Sport and Health Ethics Committee (BuSH 128) and by the Club Player Welfare officer. Participants provided written consent to take part in the study.

### Participants

Fourteen (24 years; mean weight 77.6 kg; mean height 183.2 cm) professional rugby league players from Wigan Warriors RLFC volunteered, all were participating at first team level mid competitive season.

### Experimental Protocol

This study was a randomised cross over design with participants acting as their own control. Three WBC exposures, separated by seven days following a competitive fixture the previous evening. Each WBC exposure consisted of 30 seconds precooling at −60°C then randomised (randomization.com) exposure for either 1, 2, or 3 minutes at −135°C in a liquid nitrogen cryochamber installed on a trailer (JUKA, Poland), owned and operated by BOC Linde. Prior to each WBC session, participants were prepared according to the standard BOC Linde operating protocol [16].

### Inflammatory Markers

Baseline venous blood samples were taken three days prior to the first WBC exposure. Venous blood samples were collected 20 minutes prior to and 20 minutes post WBC exposure (Table 1). All samples were collected from the antecubital vein using standard venepuncture techniques and the S-Monovette blood collection system (Sarstedt). The anticoagulant was potassium EDTA. Samples were centrifuged at 1200× g for 10 minutes and plasma was removed and stored as aliquots at −20°C until analysis. Plasma levels of interleukin six (IL-6) were analysed using a quantitative sandwich enzyme immunoassay as per manufacturer's instructions (Aviva Systems Biology), using a Perkin Elmer Enspire plate reader. All standards and samples were analysed in duplicate and interleukin six (IL-6) levels were interpolated from the standard curve using 4 parameter logistic curve fit. The immunoassay had a sensitivity to detect a concentration of at least 1 pg/mL.

**Table 1:** Timing of data collection.

Data	Base line	Pre exposure	Immediately post exposure	→5 minutes	5 minutes post exposure	→10 minutes	10 minutes post exposure	→15 minutes	15 minutes post exposure	→20 minutes	20 minutes post exposure
Core Temperature		•	•								•
Thermal Image (Tsk)		•	•		•		•		•		•
Thermal Sensation		•	•		•		•		•		•
Thermal Comfort		•	•		•		•		•		•
Blood Sample*	•	•									•
NIRO		•	•	•		•		•		•	

• = Parameter measurement protocol at each time point of testing.

* Baseline blood sampling taken three days prior to the first exposure as a normative comparison for 20 minutes post WBC exposures blood sampling.

### Tissue Oxygenation

A dual-channel continuous wave near-infrared spectrometer (NIRS) (NIRO-200 Oxygenation Monitor, Hamamatsu, Japan), with a sampling rate of 6 Hz, assessed for changes in muscle oxygenation. To evaluate muscle oxygen content, 1 emitting laser diode and 2 detecting photodiodes, absorbencies of 775, 810, and 850 nm, were used to measure the ratio of oxygenated haemoglobin to total haemoglobin [17]. Tissue oxygen saturation and haemoglobin content were determined using a modified Beer–Lambert law [18]. Measurement sites were wiped with an alcohol wipe prior to optode placement. One optode was attached to the muscle belly of the left gastrocnemius at the widest point and one optode was placed on the left vastus lateralis (VL) midway between the promixal patella and the inguinal crease. Optodes were held in place using transparent double sided hypoallergenic sticky tape and covered with a bandage. An optode spacing of 4 cm was used and the differential path length factor applied for the gastrocnemius and vastus lateralis were 4.65 and 5.99 respectively (17). Key measurements were change in oxyhaemoglobin (O2Hb); deoxyhaemoglobin (HHb) in µmol·L^−1^ from an arbitrary zero value; tissue oxygenation index (TOI) expressed as a percentage value. Data was sampled and averaged from 3×5 minute intervals pre and, every 5 minutes up to twenty minutes post WBC exposure (Table 1).

### Skin Temperature

Skin temperature (Tsk) was measured via noncontact, digital, infrared thermal imaging (TI). A ThermoVision A40M Thermal Imaging Camera (Flir systems, Danderyd, Sweden) emissivity set at 0.97–0.98 was used according to standard medical protocols [19]. The camera was mounted on a tripod height 1.2 m; distance 3.5 m from participants and connected to a laptop running Thermacam Researcher Pro 2.8 software (Flir systems, Danderyd, Sweden). To define regions of interest (ROI) wooden markers were attached mid clavicle and anterior superior iliac spine [20]. Four ROI were defined: anterior triangle of the neck (ROIA); torso (ROIB); lower abdomen (ROIC); back (ROID). Thermal images were recorded pre, immediately post and every five minutes post WBC exposure, up to 20 minutes (Table 1).

### Core Temperature

Core temperature was recorded via ingestion of a core temperature pill (CorTemp® Wireless Ingestible Temperature Sensor-Product No. HT150002. HQInc. Florida. USA.) Core temperature data was then recorded by a hand held device (CorTemp® Data Recorder-Product No. HT150001) (Table 1). Participants arrived 40 minutes prior to their allocated WBC exposure, to swallow the core temperature pill. Core temperature pills are normally swallowed 6 hours prior to measurement, however the club did not consent to this, correspondence with the pill manufacturers' confirmed that data would still be valid.

### Thermal Sensation and Thermal Comfort Questionnaires

Each participant recorded thermal sensation ratings [21] once pre, immediately following WBC and every 5 minutes up to 20 minutes (Table 1). Participants were asked ‘How are you feeling now?’ and answered by pointing to a scale from −4 to 4. (−4 = very cold, −3 = cold, −2 = cool, −1 = slightly cool, 0 = neutral, +1 = slightly warm, +2 = slightly hot +3 = hot, +4 = very hot). Thermal comfort [22] was also assessed prior to and immediately after WBC using a five point scale. Participants were asked ‘Do you find this’ and answered by choosing: 0 = comfortable; 1 = slightly comfortable; 2 = uncomfortable; 3 = very uncomfortable; 4 = extremely uncomfortable.

### Statistical Analysis

Continuous data were analysed using a mixed method model SPSS (version 19.0, SPSS Inc, Chicago, IL), using the data pre exposure as a covariate to determine changes from pre and all other time points, applying least significant difference pairwise comparisons. With the fixed factors being time points and exposure. The distributions of the data values about the mean were assessed and the data found to be suitable for parametric statistical testing. The use of an adjustment for multiple comparison was considered to be too aggressive for this exploratory study. Nominal data from the questionnaires were analysed with Friedman Tests to explore differences between all three exposures and Wilcoxon sign rank tests were used as a post hoc comparison between exposures and time points.

## Results

### Inflammatory Markers

No significant changes were recorded for the inflammatory cytokine IL-6 (Table 2).

**Table 2:** Comparison between WBC exposure times for all testing parameters and significant values.

Measurement	WBC Exposure Time (minutes)	Compared to WBC exposure time (minutes)	Mean Difference	Sig.
**Interleukin Six (IL-6) (pg/mL)**	1	2	0.37	0.81
	1	3	0.14	0.93
	2	3	−0.23	0.89
**Tissue Oxygenation Gastrocnemius HHB (µmol·L^−1^)**	1	2	−2.50	0.58
	1	3	−0.41	0.93
	2	3	2.09	0.64
**Tissue Oxygenation Gastrocnemius TOI (µmol·L^−1^)**	1	2	−0.87	0.45
	1	3	−1.98	0.08
	2	3	−1.11	0.33
**Tissue Oxygenation VL O2HB (µmol·L^−1^)**	1	2	2.24	0.37
	1	3	5.93	0.02
	2	3	3.70	0.14
**Tissue Oxygenation VL HHB (µmol·L^−1^)**	1	2	−3.82	0.14
	1	3	−2.53	0.33
	2	3	1.29	0.62
**Tissue Oxygenation VL TOI (µmol·L^−1^)**	1	2	−0.28	0.84
	1	3	1.78	0.19
	2	3	2.06	0.13
**Tsk (°C)** **	1	2	1.21	0.00
	1	3	1.70	0.00
	2	3	.50	0.00
**Core Temperature (°C)** *	1	2	0.51	0.20
	1	3	0.72	0.08
	2	3	0.20	0.62

* **Core Temperature (°C) average mean difference for all time points (pre up to 20 minutes post WBC exposure).**

** **Tsk (°C) average mean difference for all regions, for all time points (pre up to 20 minutes post WBC exposure).**

### Tissue Oxygenation

The mixed methods model showed significant reductions (p<0.05) in HHb occurred in gastrocnemius, between pre and 0–5 minutes post WBC (Table 3). Gastrocnemius HHb demonstrated a significant increase (p<0.05) between 0–5, 5–10, 10–15 minutes post WB compared to pre WBC. VL O2Hb demonstrated a significant reduction (p<0.05) when comparing a 1 minute to a 3 minute WBC exposure (Table 2). Significant differences (p<0.05) were found in HHb for VL when comparing pre WBC and 0–5 minutes post WBC and comparing 0–5 post WBC and 5–10, 10–15 minutes post WBC (Table 3). A significant reduction (p<0.05) in TOI for VL, between pre and 0–5 minutes post WBC occurred.

**Table 3:** Comparison of parameters between time points, pre, immediately post, +5, +10, +15 and +20 minutes post WBC exposures for all exposures.

Time Point*	to Time Point	Core Temp °C**	Sig.	Tsk °C***	Sig.	Blood IL-6	Sig.	NIRO G O2HB	Sig.	NIRO G HHB	Sig.	NIRO G TOI	Sig.	NIRO VL O2HB	Sig.	NIRO VL HHB	Sig.	NIRO VL TOI	Sig.
**0**	**1**	−0.50	0.21	8.85	0.00			4.28	0.31	12.79	0.03	2.36	0.11	−1.70	0.60	6.85	0.04	3.81	0.03
**0**	**2**			3.40	0.00			−2.15	0.61	−3.05	0.60	1.78	0.23	−3.74	0.25	−0.43	0.90	2.20	0.21
**0**	**3**			2.59	0.00			−1.62	0.70	−7.83	0.18	1.19	0.42	−1.80	0.58	−2.59	0.44	2.44	0.16
**0**	**4**			2.19	0.00			2.33	0.58	−6.52	0.27	0.95	0.52	−1.18	0.71	1.24	0.71	1.54	0.38
**0**	**5**	−0.38	0.35	2.01	0.00	0.15	0.91												
**1**	**2**			−5.45	0.00			−6.43	0.13	−15.85	0.01	−0.58	0.69	−2.04	0.53	−7.28	0.03	−1.61	0.35
**1**	**3**			−6.26	0.00			−5.90	0.16	−20.62	0.00	−1.17	0.43	−0.10	0.98	−9.44	0.01	−1.38	0.43
**1**	**4**			−6.66	0.00			−1.95	0.64	−19.31	0.00	−1.41	0.34	0.52	0.87	−5.61	0.10	−2.28	0.19
**1**	**5**	0.12	0.76	−6.84	0.00														
**2**	**3**			−0.81	0.00			0.53	0.90	−4.77	0.41	−0.59	0.69	1.95	0.55	−2.16	0.52	0.24	0.89
**2**	**4**			−1.21	0.00			4.49	0.29	−3.46	0.55	−0.83	0.57	2.56	0.43	1.67	0.62	−0.66	0.70
**2**	**5**			−1.39	0.00														
**3**	**4**			−0.40	0.09			3.96	0.35	1.31	0.82	−0.24	0.87	0.61	0.85	3.83	0.26	−0.90	0.60
**3**	**5**			−0.58	0.02														
**4**	**5**			−0.18	0.46														

* **Time points for all lengths of WBC exposure: 0 = pre WBC exposure, 1 = Immediately post WBC exposure, 2 = 5 minutes post WBC exposure, 3 = 10 minutes post WBC exposure, 4 = 15 minutes WBC exposure and 5 = 20 post WBC exposure.**

** **Core temperature (°C) average mean difference.**

*** **Tsk (°C) average mean difference.**

### Skin Temperature

Significant reductions (p<0.05) occurred in average Tsk when comparing 1, 2 and 3 minute WBC exposures (Table 2), when using a mixed methods model. When comparing average Tsk changes over time, significant differences (p<0.05) were found between pre exposure and all post exposure time points (Table 3). Significant differences (p<0.05) in average Tsk were found when comparing ROIC (lower abdomen) with ROI A, B and D. No significant differences were found in average Tsk between ROIB and ROID.

### Core Temperature

No significant differences were found in core temperature.

### Thermal Sensation and Thermal Comfort Scoring

The Friedman tests for the mixed method model demonstrated significant changes in thermal sensation (p = .008) and thermal comfort (p = .004) The Wilcoxson post-hoc comparison between 1 and 2 minute exposure indicated a significant reduction (p<0.05) in thermal sensation (Table 2). No significant differences were reported when comparing 1 and 3 minutes or 2 and 3 minutes. Comparison of pre and immediately post thermal sensation scores for all exposure times demonstrated significant reductions (p<0.05). No significant differences were found in thermal comfort when comparing WBC exposure times.

### Adverse Incident

On the first day of testing a Samoan player with intolerance to ice packs underwent a three minute WBC exposure. The player did not disclose his cold intolerance to the study team or BOC personnel. He suffered a mild superficial skin burn bilaterally on the mid portion of the anterior thigh. The skin damage consisting of erythema and minor blistering appeared in a horizontal strip approximately 2 cm high and 10 cm wide the day following WBC. The player was able to train normally and played competitively the following week, although willing to continue in the study, he was excluded by the study team. This adverse incident occurred despite the following safety measures. 1. The study information sheet explicitly stated that there would be an exposure to extremely cold air (−110°C to −140°C). 2. The study consent form asked about medical conditions that may affect participation. 3. The BOC screening protocol included a specific question about intolerance to cold. This incident raises an important issue, consequently we recommend that all participants receiving WBC are screened, acknowledge the contraindications of WBC and are made aware of the potential risks of exposure.

## Discussion

To our knowledge, this is the first study which has examined duration of exposure time of WBC at −135°C and effects on inflammatory marker (IL-6), tissue oxygenation, skin and core temperature, thermal sensation and comfort.

### Inflammatory Markers

Strenuous exercise is known to induce a rapid, exponential, increase in IL-6 [23]. In agreement with previous research [24] our findings revealed elevated levels of IL-6, 10–16 hours post-game. An interesting observation from our IL-6 data was the individual variability in IL-6 levels demonstrated by the high standard deviations. This variance may be explained by the lack of similarity in player movement and loading experienced during rugby league play. Several studies have shown that there are differences in player activities, and loading, as defined by position [25], [26], [27]. An appreciation of positional differences is recommended when further investigating rugby league. In the present study, a single WBC exposure, irrespective of duration, did not significantly alter IL-6 levels. This is in line with previous literature [4] investigating a 3 minute exposure, at −110°C. Our 20 minute post exposure collection point was the shortest used when compared to similar studies [7], [13], [28]. Blood sampling at 20 minutes post WBC may have been too early for any changes to have occurred in the haematological markers, and this warrants further investigation. A previous study [28] reported that blood samples were taken 30 minutes post 3 minute WBC exposure, with no reports of inability to collect blood samples. An interesting challenge to the collection of venous blood samples was the partial ‘shutdown’ of the peripheral vasculature, particularly the median veins of the antecubital fossa. It appeared that vascular shunting from upper to lower limbs took place especially following 3 minute WBC exposure. Despite the target veins being visibly distended, as expected in very lean, muscular athletes; venous blood flow was severely impaired in several participants. Venepuncture was successful in all cases, yet an appropriate level of peripheral blood flow was often compromised, particularly following the 3 minute WBC exposure. This led to incomplete inflammatory myokine profiles being generated for some participants.

### Tissue Oxygenation

**Figure 1:**
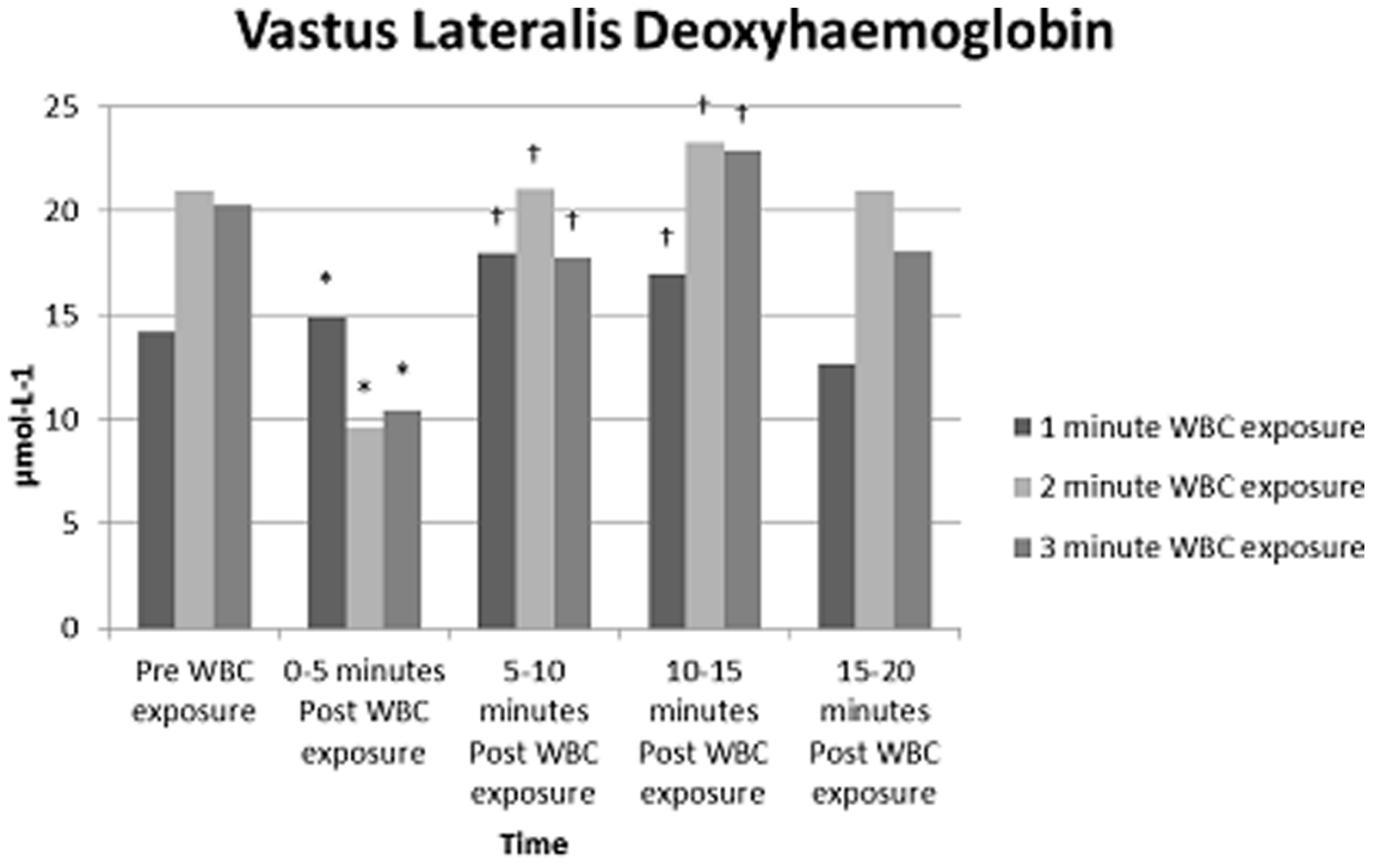
Mean NIRO values for VL, pre and post WBC exposure for all three WBC time exposures (1, 2 and 3 minutes). Values are means (N = 9). Statistical significance (P<0.05) observed over a period of time (pre, immediately post exposure, +5 minutes, +10 Minutes and, +15 minutes post exposure. * indicates significantly different to pre WBC; †indicates significantly different to 0–5 min post WBC.

**Figure 2:**
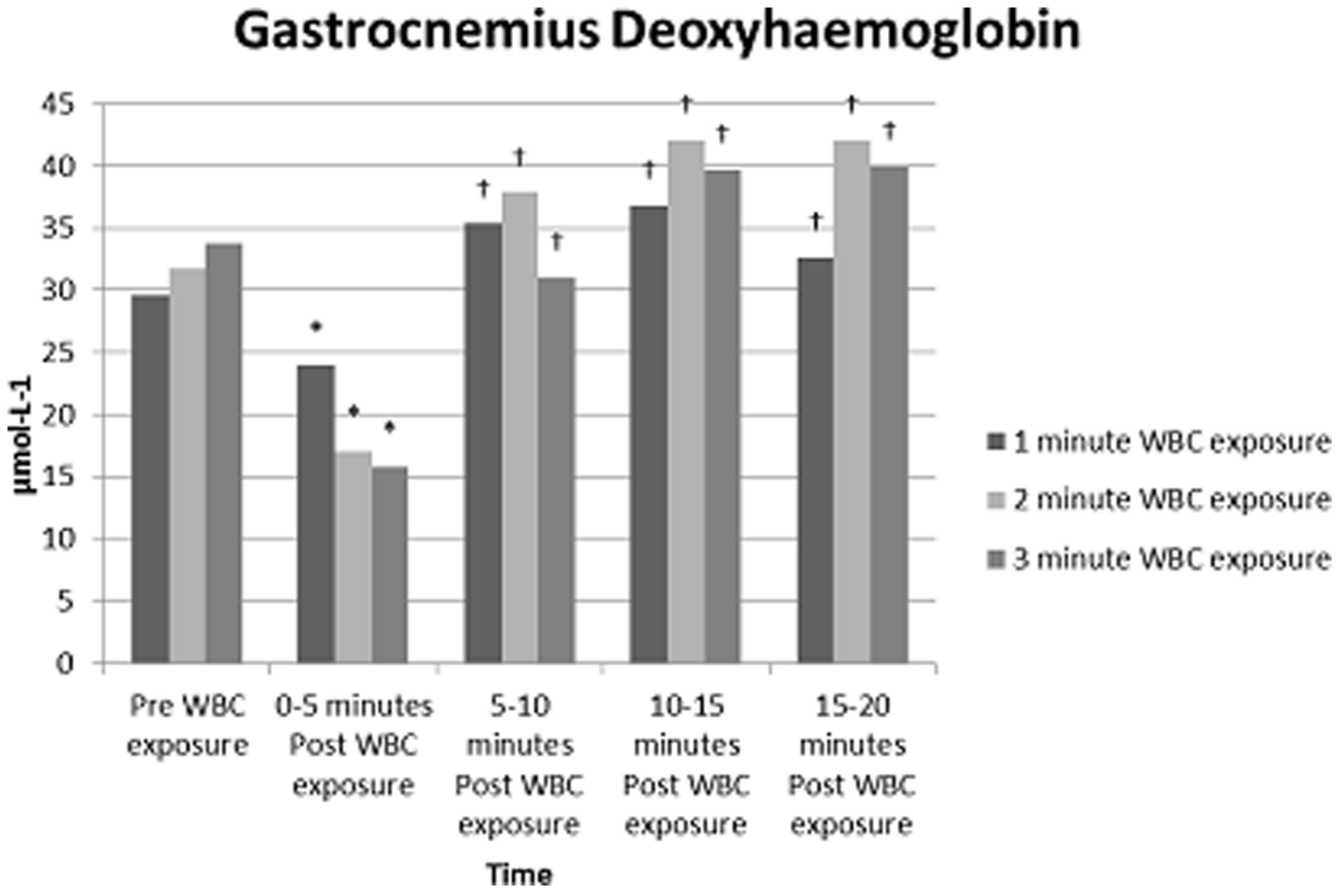
Tissue deoxygenation for gastrocnemius, pre and post WBC exposure for all three WBC time exposures (1, 2 and 3 minutes). Values are means (N = 9). Statistical significance (P<0.05) observed over a period of time (pre, immediately post exposure, +5 minutes, +10 Minutes, +15 minutes and +20 minutes post exposure. * indicates significantly different to pre WBC; †indicates significantly different to 0–5 min post WBC.

NIRO data demonstrate that a 3 minute WBC exposure differed significantly to a 1 or 2 minute WBC exposure. Results suggest localised tissue hypoxia in VL occurred as O2Hb decreased with a subsequent increase in HHb. (Figure 1). In gastrocnemius, a decrease in blood volume, 0–5 minutes post WBC occurred (Figure 2). O2Hb and HHb both increased to above pre WBC exposure measures over the remaining 15 minutes, indicative of venous pooling. These findings are supported by previous research that suggests extreme cold exposure reduces cardiac output and therefore produces a shift in blood volume to the venous circulation leading to pooling and reduced venous return [29]. Results indicate that gastrocnemius was more susceptible to pooling at all exposures than VL, possibly due to a weaker vasoconstriction response rather than active vasodilation. Vasoconstriction of peripheral blood vessels to maintain core temperature is generally observed with moderate cold exposure [30]. Initial responses during the present study demonstrate this physiological response. However, under extreme cold exposure the body endeavours to protect the extremities. As deeper muscles lose heat to the surface, cold-induced vasodilation (CIVD) can occur [31]. The exact mechanisms leading to CIVD are not fully understood. However, an increase in peripheral blood flow/volume followed by pooling of the blood has been observed [32] as was evident following a 3 minute WBC exposure in the present study. CIVD is thought to initiate when core temperatures rise above 36°C [31]. In the current study, core temperature changes although insignificant demonstrate a rise to 37°C immediately post WBC, this would potentially lend support to the concept of CIVD response. Although supporting previous literature [33], caution is needed when using CIVD as an explanation of our data as we did not have continuous data on blood flow, Tsk or core temperature.

NIRO is sensitive to small movements and as a result of participants standing for 10 minutes pre and 20 minutes post WBC, some contraction of the muscles would have occurred. It is impossible to avoid movement, however some participants may have moved around more than others, affecting the results. Future research should take this in to account and have participants resting on a plinth.

### Skin and Core Temperature

**Figure 3:**
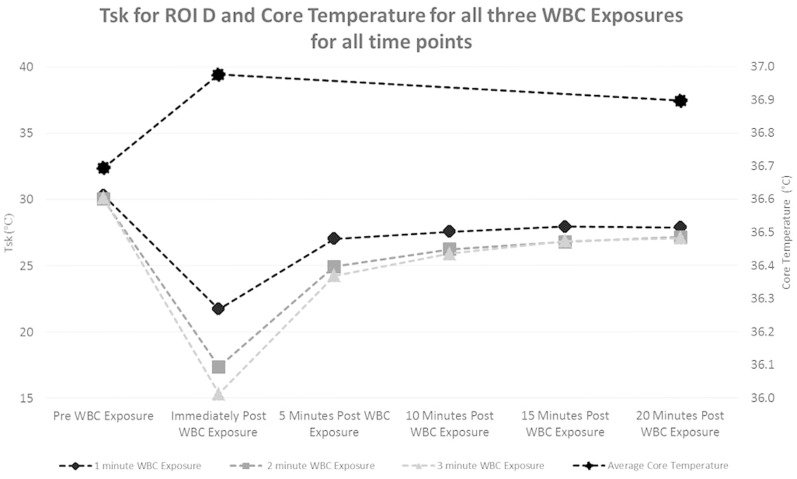
Core and Abdomen skin temperature before and after WBC exposure (1, 2 and 3 minutes). Y-axis on the left measures skin temperature and Y-axis on the right measures core temperature. Values are means (N = 9). for timepoints, pre, immediately post exposure, +5 minutes, +10 Minutes, +15 minutes and +20 minutes post exposure.

All exposure times of WBC reduced Tsk in all four ROI. Previous studies have shown the effectiveness of WBC in the reduction of Tsk [21]. The lowest Tsk recorded in the current study was 12.1°C in two out of the nine participants, following a three minute exposure in ROI ‘C’ (lower abdomen). As reported in previous studies, an extended phase of rewarming occurred following WBC exposure, no significant changes in Tsk occurred beyond 10 minutes post exposure and mean Tsk at 20 minutes post WBC did not reach mean Tsk pre WBC [34], [35] (Figure 3). Although core temperature did not show any significant changes following WBC, core temperature displays a predictable relationship with Tsk; where the maximum post WBC drop in Tsk occurred, a small rise in core temperature was observed for all exposure times (Figure 3). The core temperature pill was ingested 40 minutes prior to WBC exposure and although a longer time is normal, results did demonstrate change in core measurement temperatures. The pattern of change in Tsk and the slight rise in core temperature suggests vascular shunting to maintain the functions of vital organs.

### Thermal Sensation and Thermal Comfort Scoring

Prior to WBC participants rated their thermal sensation as ‘neutral’ or ‘slightly warm’. Following all WBC exposure times significant reductions in thermal sensation were noted, in most cases participants rated themselves as ‘very cold’ or ‘cold’. No significant differences occurred between 2 and 3 minutes exposures for thermal sensation, however between 1 and 2 minute exposures there was a significant reduction (p<0.05) in thermal sensation scores. Immediately post 1 minute WBC exposure participants reported feeling ‘slightly cool’ compared 2 minutes where participants reported feeling ‘cold’ or ‘very cold’. The differences in perceived temperature sensation suggest that 1 minute may not be a long enough WBC exposure to initiate perceptual changes in temperature sensation.

### Limitations

**Figure 4:**
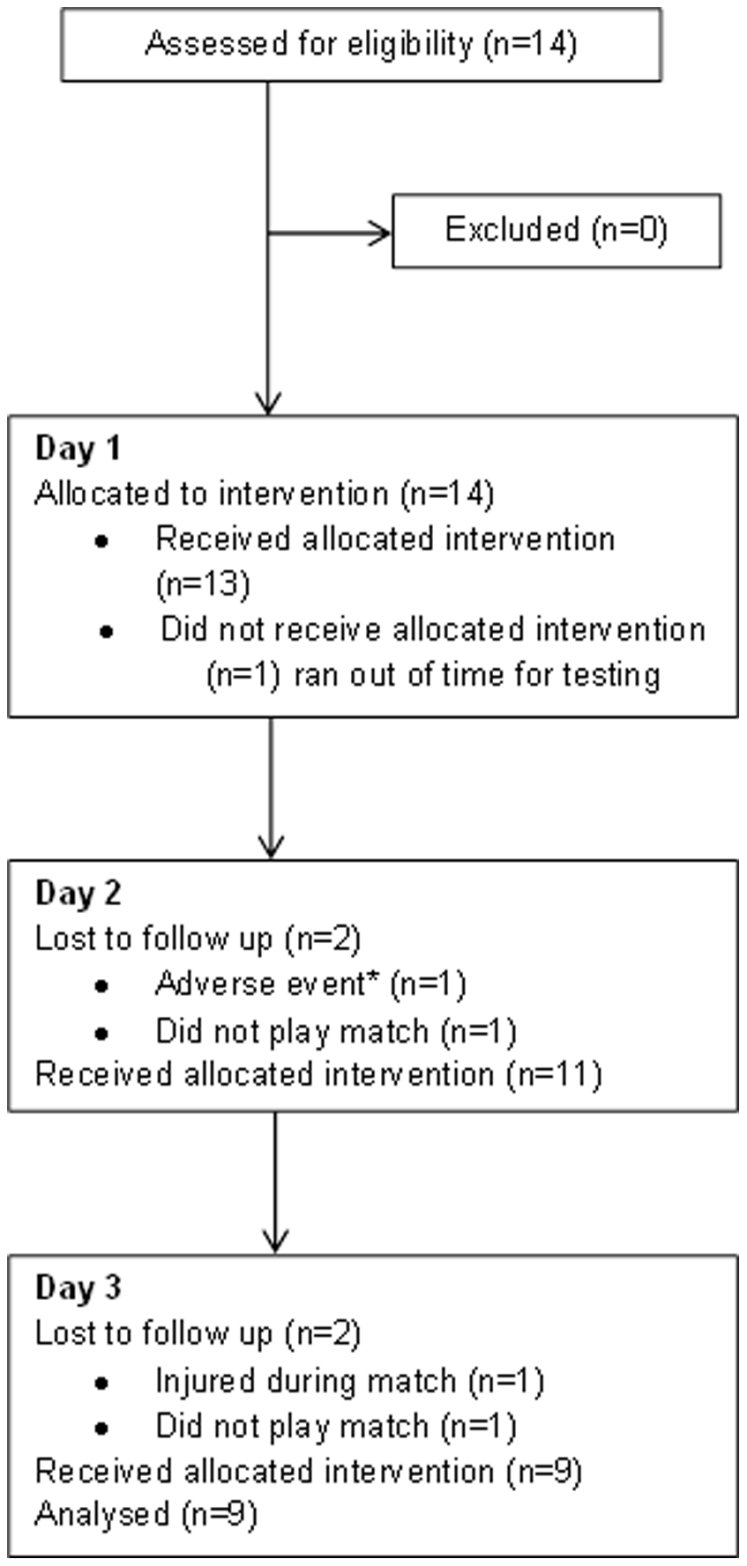
CONSORT diagram showing participant flow and retention.

Although ecologically valid, with elite players participating in competitive fixtures mid-season, there was potential for players to drop out due to injury or non-selection to play (Figure 4). The training ground environment in which the testing took place was also a limitation, as a temperature controlled room was not available. We also acknowledge that although the temperature of the cryochamber was set at −135°C, fluctuations occurred during the exposure times, which is common with prolonged use and any thermal stimulation [36]. No skin surface temperatures were measured during WBC exposure periods neither was air flow, in order to observe convective strength of WBC. This was due to the lack of equipment suitable to measure such parameters. We were unable to measure internal recommended temperature as reported by Savic et al (2013) therefore differences in actual and reported temperatures may vary. The core temperature pill should ideally be ingested approximately 6 hours prior to testing, future studies should take this into consideration.

### Summary

A pattern in the results from this study emerged suggesting that a 2 minute WBC exposure at −135°C following 30 seconds pre-cooling at −60°C was the optimum WBC exposure for first team professional rugby league players. A 2 minute WBC exposure induces potentially beneficial physiological and perceptual changes, greater than those achieved following a 1 minute WBC exposure but without any of the negative effects demonstrated by a 3 minute exposure. Whilst we have not demonstrated support for a single bout of WBC, other studies have shown the potential of WBC in mediating muscular recovery by attenuating the inflammatory process [4]. Successful use of WBC to ‘blunt’ the inflammatory response appears to be effective when administered immediately post exercise, with repeat exposures. This supports recent literature, suggesting that physiological changes are dependent on the number of sessions of WBC [28]. It is therefore suggested that future research could follow a similar protocol to this study, using a 2 minute WBC exposure at −135°C this would facilitate the development of a stronger evidence base for WBC. These studies should focus on determining the optimum number of sessions per day/week and also over what time period exposure cycles should take place.

### What are the new findings?

- 30 seconds at −60°C followed by 2 minutes WBC at around −135°C appear to be an optimum WBC exposure time.
- 2 minute WBC exposure produces physiological changes in core and skin temperature, tissue oxygenation in vastus lateralis and gastrocnemius muscles and thermal sensation responses.
- Professions working within elite sport can be advised on applying an optimal WBC exposure time of 2 minutes at around −135°C for physiological changes to occur.

### How might it impact on clinical practice in the near future?

- A duration time of 2 minutes of WBC exposure at −135°C has been established as a safe protocol for future application for male elite rugby league players.
- Clinicians should utilise the protocol from this study to compare future research which will help now determine the number of required WBC exposure sessions to initiate a larger physiological responses.
- Research investigating WBC on other elite athletes and women would be advantageous to broaden the knowledge on WBC across a variety of sports and gender.
